# IgG Fc galactosylation predicts response to methotrexate in early rheumatoid arthritis

**DOI:** 10.1186/s13075-017-1389-7

**Published:** 2017-08-09

**Authors:** Susanna L. Lundström, Aase H. Hensvold, Dorothea Rutishauser, Lars Klareskog, A. Jimmy Ytterberg, Roman A. Zubarev, Anca I. Catrina

**Affiliations:** 1 Division of Physiological Chemistry I, Department of Medical Biochemistry and Biophysics, Karolinska Institutet, Scheelesväg 2, SE 17177 Stockholm, Sweden; 2 Rheumatology Unit, Department of Medicine, Karolinska Institutet, Stockholm, Sweden; 3 Rheumatology Unit, Karolinska University Hospital, Stockholm, Sweden

**Keywords:** Immunoglobulin, Glycosylation, Rheumatoid arthritis, Methotrexate, Complement, Biomarker

## Abstract

**Background:**

Methotrexate (MTX) is the standard first-line therapy in rheumatoid arthritis (RA) with variable clinical efficacy that is difficult to predict. The glycosylation status of immunoglobulin G (IgG) is altered in RA and influenced by MTX treatment. We aimed to further investigate if IgG glycosylation in untreated early RA can predict therapeutic response to MTX.

**Methods:**

We used a shotgun proteomic approach to screen for the Fc glycopeptides in the serum of 12 control subjects and 59 untreated patients with early RA prior to and following MTX initiation. MTX treatment response was defined according to the European League Against Rheumatism at a median of 14 weeks (range 13–15) after treatment initiation. Seropositive patients were defined as those testing positive for anticitrullinated protein antibodies and/or rheumatoid factor at baseline (*n* = 44). Data analysis was performed using uni- and multivariate statistics.

**Results:**

We could confirm a low abundance of galactosylated glycans in untreated patients with early RA compared with control subjects that was partially restored by MTX treatment. This was more evident among future nonresponders than among responders to MTX treatment. Results were further validated and confirmed by multivariate statistical analysis of the baseline Fc glycan, proteomic, and clinical data. We found that the ratio between the main agalactosylated (FA2) and main mono- and di-galactosylated Fc glycans (FA2G1 and FA2G2) of IgG1 ranked as the most prominent factor distinguishing responders from nonresponders. A low baseline ratio of FA2/[FA2G1 + FA2G2]-IgG1 was associated with nonresponse (OR 5.3 [1.6–17.0]) and was able to discriminate future nonresponders from responders to MTX therapy with a sensitivity of 70% (95% CI 46–88%) and a specificity of 69% (95% CI 52–83%). For seropositive patients (*n* = 44), this trend was improved with a sensitivity of 73% (95% CI 45–92%) for nonresponse and a specificity of 79% (95% CI 60–92%).

**Conclusions:**

We show that the FA2/[FA2G1 + FA2G2] of IgG1 is a biomarker candidate that is significantly associated with nonresponding patients and has potential value for prediction of MTX clinical response.

**Electronic supplementary material:**

The online version of this article (doi:10.1186/s13075-017-1389-7) contains supplementary material, which is available to authorized users.

## Background

Rheumatoid arthritis (RA) is an autoimmune disease in which autoantibodies, and especially anticitrullinated protein antibodies (ACPA) and rheumatoid factor (RF), are thought to play an important pathogenic role. Effector functions of antibodies are mediated largely through the fragment crystallizable (Fc) portion, such as phagocytosis; cell cytotoxicity; complement activation; and, more recently, osteoclast activation [1, 2]. In general, these functions are dependent on the glycan structures present on the constant heavy chains of the Fc portion of immunoglobulin G (IgG). The more complex the glycan is (particularly if the glycan contains galactose and sialic acid), the less likely it is for antibodies to have a proinflammatory effect [3–6]. For this reason, it has been suggested that the amount of galactosylated glycans, or the ratio between agalactosylated and galactosylated Fc glycans, is an indicator of inflammation and immune system activation [7]. In RA, agalactosylated and asialylated glycans are prominent, and the presence of these modified glycans often correlates with disease severity and progression [8–10]. Furthermore, ACPA IgGs have a perturbed pattern of Fc glycans compared with the total IgG pool [10–12].

Methotrexate (MTX) is the standard first-line therapy in RA with a varying clinical efficacy that is difficult to predict [13–15]. Several studies have suggested that MTX treatment results in significantly increased numbers of Fc-galactosylated glycans, as demonstrated in several small long-standing RA cohorts [16–18].

The aim of the present study was to investigate IgG Fc glycan structures in untreated early RA and their role as biomarkers for response to MTX treatment, using a sensitive and IgG isotype-specific shotgun proteomic approach. By using this approach, we could simultaneously measure the levels of specific proteins of interest (such as the complement pathway proteins and the IgG1–IgG4 protein isotypes). Via multivariate analysis, we could then investigate how these proteins relate to the observed Fc glycan distribution profile.

Our results indicate a general low abundance of galactosylated glycans in untreated early RA and that this feature is partially restored by MTX treatment. Furthermore, the galactosylation status of the IgG-Fc, and specifically the ratio between the main agalactosylated and mono- and di-galactosylated Fc glycans of IgG1_,_ (FA2/[FA2G1 + FA2G2]), was shown to be the best overall biomarker candidate that is predictive of therapeutic response.

## Methods

### Patients

**Fig. 1:**
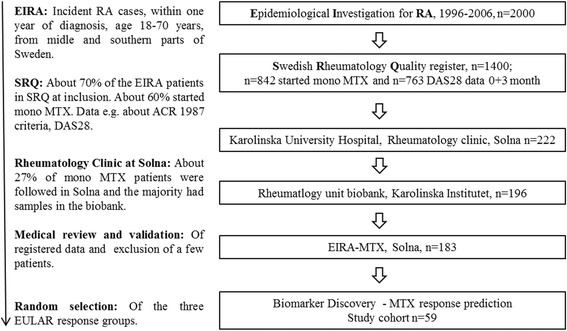
Overview of study recruitment and data collection. ACR American College of Rheumatology, DAS28 Disease Activity Score in 28 joints, EULAR European League Against Rheumatism, MTX Methotrexate

This study was performed with a cohort of 59 untreated patients with early RA recruited during 1996–2006 at the rheumatology clinic at Karolinska University Hospital, Stockholm, Sweden. The selected cohort is part of the nationwide Epidemiological Investigation of Rheumatoid Arthritis (EIRA) cohort [19, 20], whose demographic characteristics are described in Additional file 1: Table S1. The patients were in the stage of untreated early RA with symptom onset <1 year prior to diagnosis. Serum sample selection was based on availability and to give a representation clinically similar to the larger EIRA cohort. The control subjects were similarly selected from among the healthy control subjects originally included in the EIRA control cohort according to age, sex, and geographical area matched to the RA patient study group. Samples were obtained at baseline and at clinical follow-up, which occurred after a median of 14 weeks (25–75% IQR 13–15). All patients started on MTX, with or without concomitant nonsteroidal anti-inflammatory drugs and/or prednisolone, to a final dose of 10–20 mg/week following local guidelines. Seropositive patients were defined as those testing positive for ACPA and/or RF at baseline (*n* = 44). An overview of recruitment and data collection is presented in Fig. 1. Response to MTX was categorized according to European League Against Rheumatism (EULAR) response criteria [21, 22] at follow-up examinations. Patients with no response to MTX were compared with patients with good and moderate responses to MTX. Additionally, serum samples from 11 healthy individuals who were matched to the patient group by age and sex were analyzed. Ethical approval for this work was obtained from the regional ethical review board (96-174[1996-0419] and 2006-476-31/4), Karolinska Institute, Stockholm, Sweden. An informed consent form (as documented by caregivers in patient records) was given to all participants as specified in the ethical approval and in line with Swedish law. Demographic characteristics of the patients and control subjects are summarized in Table 1.

**Table 1:** Characteristics and of patients and healthy control subjects

	Healthy control subjects (*n* = 11)	Early RA				
		All patients (*n* = 59)	GR (*n* = 19)	MR (*n* = 20)	NR (*n* = 20)	*p* Value^a^
Age, years	54 (45–57)	53 (45–62)	49 (41–63)	49 (41–56)	55 (49–65)	0.03
Female sex	73%	72%	68%	65%	80%	0.28
Current smoking	10%	34%	31%	30%	30%	0.52
ACPA- and/or RF-positive	–	75%	74%	75%	75%	0.96
DAS28-ESR	–	5.7 (5.0–6.2)	5.2 (5.0–6.0)	6.0 (5.4–6.4)	5.2 (4.7–6.4)	0.40
HAQ	–	1.3 (0.9–1.6)	1.1 (0.8–1.4)	1.4 (1.0–1.9)	1.3 (0.9–1.6)	0.66
DAS28-ESR follow-up	–	4.5 (2.6–5.1)	2.2 (1.7–2.6)	4.4 (4.0–4.9)	5.4 (4.9–5.8)	<0.0001
MTX duration, weeks	–	14 (13–15)	14 (13–15)	14 (13–15)	14 (14–15)	0.50
Prednisolone	–	25%	31%	30%	15%	0.19

*Abbreviations: ACPA* Anticitrullinated protein antibodies, *DAS28-ESR* Disease Activity Score in 28 joints based on erythrocyte sedimentation rate, *GR* Good response, *HAQ* Health Assessment Questionnaire, *MR* Moderate response, *MTX* Methotrexate, *NR* No response, *RA* Rheumatoid arthritis, *RF* Rheumatoid factor

All characteristics are from assessments at baseline, except when otherwise stated. Values are of the median (first to third quartiles), except when otherwise indicated. Response was categorized according to European League Against Rheumatism response criteria

^a^Comparison of patients not responding (no European League Against Rheumatism [EULAR] response) and patients responding (EULAR good or moderate response)

### Sample preparation and analysis

Samples were treated similarly to what has previously been described [23]. Ten micrograms of serum protein were preincubated in 0.3% ProteaseMAX (Promega, Madison, WI, USA) and 3 M urea (30 minutes, 50 °C), followed by bath sonication (for 10 minutes, 20 °C). The samples were diluted three times, and acetonitrile was added to a final concentration of 10% [23]. The proteins were reduced (5 mM dithiothreitol, 30 minutes, 56 °C) and alkylated (14 mM iodoacetamide, 30 minutes in darkness). Trypsin was added at a ratio of 1:50 (enzyme to protein) for overnight digestion at 37 °C. Peptides were desalted using C18 StageTips (Thermo Fisher Scientific, West Palm Beach, FL, USA), dried, and resuspended in 0.2% formic acid and 3% acetonitrile prior to analysis. Samples were kept at 10 °C, injected into columns in 1-μg aliquots, and analyzed in randomized order using a reversed-phase liquid chromatography system (EASY-nLC) connected to a Q Exactive Orbitrap mass spectrometer (MS; both from Thermo Fisher Scientific). The MS was operating in positive ion mode, and the survey mass spectrum was obtained in the mass-to-charge ratio range of 300–2000 with a nominal resolution of 60,000. Following each survey mass spectrum, the five most abundant precursor ions were selected for and subjected to tandem mass spectrometry (MS/MS) using higher-energy collisional dissociation fragmentation.

### Fc glycopeptide identification and quantification

As previously described [11, 23], we screened for 19 different glycoforms N-linked to the tryptic peptides EEQYNSTYR and TKPREEQYNSTYR (IgG1), EEQFNSTFR and TKPREEQFNSTFR (IgG2), as well as EEQFNSTYR/EEQYNSTFR and TKPREEQFNSTYR/TKPREEQYNSTFR (IgG3 and IgG4). Thus, in total, 114 glycopeptides were screened for. Because each glycan substitution site was quantified by two peptides, this corresponds to 57 Fc glycan variants. Quantification was performed in a label-free manner using the Quanti program [24]. Glycopeptide ion abundances were integrated over the respective chromatographic peaks of monoisotopic ions (<10 ppm deviation from the theoretical mass value) with the charged states described above and within a ±2-minute interval around the expected retention times as determined by human polyclonal IgG standard (Sigma-Aldrich, St. Louis, MO, USA). Glycan abundances were normalized to total content of Fc-glycosylated IgG1 or IgG2 peptides. Extracted ion chromatograms of samples of a patient with RA and a control subject are shown in Additional file 1: Figure S1.

### Fc protein isotype and complement protein identification and quantification

Protein identification and quantification were obtained similarly to what has previously been described [11, 23]. Complement pathway proteins were normalized by the logarithmic value of the total intensity of unique peptides for the respective protein. The relative intensity of unique Fc peptides from IgG1, IgG2, IgG3, and IgG4 were used to calculate the IgG isotype distribution. A list of the number of peptides and scores used for identification/quantification of the complement proteins and IgG isotypes is given in Additional file 1: Table S2.

### Statistical analysis

Univariate statistical analysis was performed using Student’s *t* test (with equal or unequal variance depending upon the *F*-test) for interindividual variation and with Student’s paired *t* test for intraindividual variation, respectively. Because the majority of the less significant *p* values (i.e., 0.05–0.01) are confirmed to be more prominent for nonresponders (interindividually) or good responders (intraindividually), we chose to show the uncorrected values to easily point out these trends. False discovery rate (FDR) correction of *p* values was performed according to the total number of comparisons (*n* = 522) in the study. *p* Values <0.02 (*n* = 130) remained significant following the correction. ROC curve analysis was performed using Prism version 5.02 for Windows software (GraphPad Software, La Jolla, CA, USA). Sensitivity and specificity were estimated by 2 × 2 contingency tables using SAS version 9.3 software (SAS Institute, Cary, NC, USA). Principal component analysis (PCA) and orthogonal projections to latent structures discriminant analysis (OPLS-DA) were performed using SIMCA 14.0 (Umetrics, Umeå, Sweden) following mean centering, logarithmic transformation, and univariate scaling. Model performance was reported as cumulative correlation coefficients for the model (*R* ^2^ × [cum]), with predictive performance being based on sevenfold cross-validation calculations (*Q* ^2^[cum]) and analysis of variance of cross-validated residuals (CV-ANOVA) *p* values.

## Results

### A high agalactosylated/galactosylated Fc glycan ratio is present in untreated early RA

**Fig. 2:**
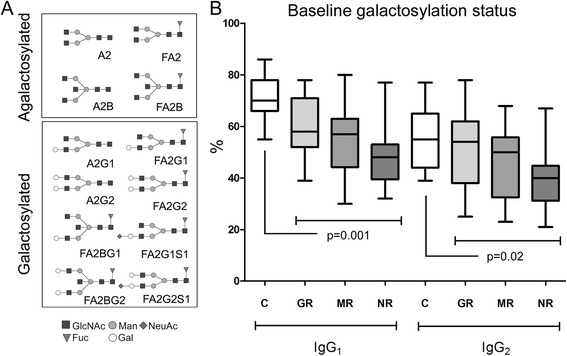
Distribution of galactosylated immunoglobulin G1 (IgG1)- and IgG2-fragment crystallizable (Fc) glycans. a Quantified glycan structures grouped as agalactosylated, (aGal) and galactosylated (Gal) glycans. Glycan nomenclature is according to Royle et al. [35]. The core oligosaccharide structure comprises a biantennary heptasaccharide moiety (A2). Usually, the first sugar unit (an N-acetylglucosamine) is additionally core-fucosylated (e.g., FA2). The biantennary structure can also be transected by an additional N-acetylglucosamine (FA2B). Furthermore, the outer glucosamine units can be elongated with galactoses (FA2Gn, n = 1 or 2), and the galactoses can be further extended with sialic acids (FA2GnSn, n = 1 or 2). b Distribution of galactosylated IgG1- and IgG2-Fc glycans at baseline in healthy control subjects and in patients with early rheumatoid arthritis (RA). The relative distribution (%) of galactosylated Fc glycans differed significantly (p < 0.05) in healthy control subjects compared with patients with early RA and when comparing patients with good response and no response. Collectively, these data demonstrate a changed galactosylation pattern of Fc glycans in RA, with lower proportions of galactosylated glycans than their agalactosylated counterparts. The effect is most pronounced in IgG1. Following false discovery rate correction, p = 0.001 remains significant. C Complement protein, MR Moderate response

Of the 57 Fc glycopeptides we screened for (based on pure standard IgG), 12 Fc glycans from IgG1 and 7 from IgG2 were profiled directly in the trypsin-digested serum samples (Additional file 1: Table S3). Identified glycan structures are shown in Fig. 2a. When comparing patients with untreated RA with control subjects, it was evident that the Fc glycan profile of RA was significantly (*p* < 0.05) altered: Of the 19 profiled glycans, 12 (8 from IgG1 and 4 from IgG2) were significantly different (Additional file 1: Table S4).

To test the influence that the different Fc glycan sugar types have on the RA patient profile and their potential as biomarkers for response to treatment, the IgG1- and IgG2-Fc glycans were grouped as galactosylated (containing galactose [G]), agalactosylated (lacking galactose [aG]), afucosylated (lacking fucose [aF]), bisected (containing glycan bisection [B]), and sialylated (containing sialic acid [S]). Similarly to what has previously been reported [25, 26], among the Fc glycans, the agalactosylated forms were in general more abundant (*p* < 0.05), whereas the galactosylated forms were less abundant (*p* < 0.05) in patients with early RA as than in healthy control subjects (Fig. 2b and Additional file 1: Table S5). Noteworthy among sialylated and afucosylated glycans (which have been reported as perturbed in RA pathogenesis [27, 28]) is that no significant differences were observed between patients with RA and control subjects. Interestingly, significantly lower abundance of the bisected forms of IgG2 was observed.

### MTX partially restores IgG-Fc glycan profiles in early RA

MTX treatment made the early RA Fc glycan profile more similar to that of the control subjects (Additional file 1: Tables S4 and S6). More specifically, we observed in responding patients a significant intraindividual decline in the agalactosylated IgG1 glycans, an increase of the galactosylated glycans particularly in IgG1, and an increase in bisected glycans of IgG2 (Table 2). However, this intraindividual shift was not evident in patients with no response to treatment (Table 2, Additional file 1: Figure S2). Similarly, a significant decline in the ratio between the agalactosylated and galactosylated (aGal/Gal) glycans was observed in the patients responding to MTX but not in the nonresponding patients (Table 2).

**Table 2:** Immunoglobulin G fragment crystallizable glycan distribution of IgG1 and IgG2 in patients with rheumatoid arthritis prior to and following methotrexate treatment

Type	Isotype	Glycan^a^	Good responders		Moderate responders		Non responders		Intraindividual change			Interindividual differences	
									Prior/following			Prior	Following
			Prior	Following	Prior	Following	Prior	Following	GR	MR	NR	(GR + MR)/NR	(GR + MR)/NR
	IgG1	Σ[Galactosylated glycans]^b^	60 ± 12	63 ± 13	54 ± 13	57 ± 13	49 ± 12	47 ± 14	0.004	0.03	0.5	0.02	0.001
		Σ[Agalactosylated glycans]^c^	40 ± 12	37 ± 13	46 ± 13	43 ± 13	51 ± 12	53 ± 14	0.004	0.03	0.5	0.02	0.001
		Σ[Afucosylated glycans]^d^	4 ± 3	4 ± 3	3 ± 2	4 ± 2	3 ± 1	4 ± 2	0.8	0.4	0.2	0.2	1
		Σ[Bisected glycans]^e^	12 ± 4	13 ± 4	12 ± 2	14 ± 3	13 ± 3	14 ± 5	0.2	0.0003	0.3	0.7	1
		Σ[Sialylated glycans]^f^	4 ± 4	4 ± 3	3 ± 2	4 ± 3	2 ± 2	3 ± 2	0.5	0.3	0.5	0.1	0.04
	IgG2	Σ[Galactosylated glycans]^g^	52 ± 14	54 ± 15	45 ± 13	47 ± 13	40 ± 11	39 ± 11	0.2	0.1	0.7	0.01	0.003
		Σ[Agalactosylated glycans]^h^	48 ± 14	46 ± 15	55 ± 13	53 ± 13	60 ± 11	61 ± 11	0.2	0.1	0.7	0.01	0.003
		Σ[Bisected glycans]^i^	7 ± 3	8 ± 3	6 ± 2	7 ± 3	7 ± 3	7 ± 3	0.01	0.004	1	0.7	0.5
		Σ[Sialylated glycans]^j^	6 ± 5	6 ± 5	5 ± 3	5 ± 4	4 ± 2	4 ± 2	0.5	0.9	0.9	0.02	0.1
aGal/Gal ratio	All IgG1	Log [Σ(Agalactosylated glycans)/Σ(galactosylated glycans)]	−0.18 ± 0.24	−0.25 ± 0.26	−0.08 ± 0.25	−0.14 ± 0.25	0.02 ± 0.21	0.05 ± 0.26	0.003	0.03	0.42	0.03	0.006
	All IgG2	Log [Σ(agalactosylated glycans)/Σ(galactosylated glycans)]	−0.05 ± 0.26	−0.07 ± 0.27	0.09 ± 0.24	0.05 ± 0.24	0.19 ± 0.21	0.20 ± 0.22	0.2	0.05	0.62	0.007	0.006
	Main IgG1	Log[FA2/(FA2G1 + FA2G2)]	−0.17 ± 0.21	−0.24 ± 0.23	−0.07 ± 0.23	−0.13 ± 0.22	0.02 ± 0.20	0.05 ± 0.24	0.001	0.02	0.4	0.02	0.001
	Main IgG2	Log[FA2/(FA2G1 + FA2G2)]	0.02 ± 0.23	−0.05 ± 0.24	0.11 ± 0.23	0.06 ± 0.21	0.20 ± 0.21	0.22 ± 0.20	0.1	0.02	0.6	0.02	0.001
	Bisected IgG1	Log[FA2B/(FA2BG1 + FA2BG2)]	−0.15 ± 0.29	−0.21 ± 0.29	−0.08 ± 0.26	−0.12 ± 0.25	0.06 ± 0.24	0.07 ± 0.26	0.03	0.1	0.7	0.02	0.002
	Bisected IgG2	Log[FA2B/FA2BG1]	0.40 ± 0.35	0.35 ± 0.42	0.68 ± 0.48	0.52 ± 0.46	0.76 ± 0.38	0.70 ± 0.29	0.4	0.03	0.3	0.1	0.02
	Afucosylated IgG1	Log[A2/(A2G1 + A2G2)]	−0.51 ± 0.62	−0.72 ± 0.71	−0.36 ± 0.59	−0.53 ± 0.58	−0.17 ± 0.56	−0.19 ± 0.56	0.02	0.04	0.8	0.1	0.01

*Abbreviations*: *aGal/Gal* Ratio between agalactosylated and galactosylated glycans, *GR* Good response, *MR* Moderate response, *NR* No response

Patients are grouped according to response to MTX. Figures are given for average values (%) ± SD. *p* Values <0.02 remained significant following false discovery rate correction

^a^Glycan abbreviations are provided in Fig. 2a legend

^b^Σ[IgG1: A2G1, A2G2, FA2G1, FA2BG1, FA2G1S1, FA2G2, FA2BG2, FA2G2S1]

^c^Σ[IgG1: FA2, FA2B, A2 and A2B]

^d^Σ[IgG1: A2, A2B, A2G1, A2G2]

^e^Σ[IgG1: A2B, FA2B, FA2BG1, FA2BG2]

^f^Σ[IgG1: FA2G1S1, FA2G2S1]

^g^Σ[IgG2: FA2G1, FA2BG1, FA2G2, FA2G1S1, FA2G2, FA2G2S1]

^h^Σ[IgG2: FA2, FA2B]

^i^Σ[IgG2: FA2B, FA2BG1]

^j^Σ[IgG2: FA2G1S1, FA2G2S1]

We further tested if other structural variations in the Fc glycan would affect the aGal/Gal ratio. The results (Table 2, Additional file 1: Figure S3) indicated a similar trend in all galactosylated species (i.e., independent of other glycan substitution patterns, such as *N*-acetylglucosamine bisection or fucosylation to the inner core unit). Similarly, even though IgG2 contained less Fc-galactosylated species than IgG1 in all tested individuals (Fig. 2b), the aGal/Gal ratios correlated between IgG1 and IgG2 relatively well intraindividually (Additional file 1: Figure S3), thus indicating that in patients with RA, both IgG1 and IgG2 contain lower amounts of Fc-galactosylated species than what is normal.

### Baseline Fc galactosylation status ranks high in distinguishing responders and nonresponders in a multivariate model

**Fig. 3:**
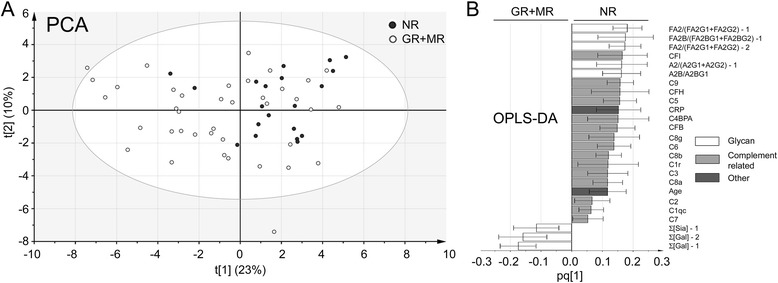
Multivariate model based on baseline information of the patients prior to methotrexate treatment. a Principal component analysis (PCA) score plot. The majority of patients with no response to treatment (NR, dark circles) cluster together along component 1 (x-axis). This indicates that there are factors at baseline that are characteristic for this patient group within the model. b The orthogonal projections to latent structures discriminant analysis (OPLS-DA) loading plot of the same data reveals which factors these are. The error bars represent the range of each marker’s value (with >95% confidence). Features that are positive correlate with no response, features that are negative correlate with response. Shown are only the features correlating with 95% confidence. For details of the complete model, see Additional file 1: Table S7. C Complement protein, CF Complement factor, FA2 Main agalactosylated Fc glycan, FA2G1 Main monogalactosylated Fc glycan, FA2G2 Main digalactosylated Fc glycan, GR Good response, MR Moderate response, NR No response

Multivariate statistical modeling was done to find out how well the baseline levels of the Fc glycans would correlate with other variables and reflect the response (or absence thereof) to treatment. Other biomarker candidates included in the analysis were proteins involved in the complement pathways and the IgG1–IgG4 protein isotypes that were present in the proteomic analysis (i.e., acquired simultaneously with the Fc glycans in the analyses of sera by liquid chromatography-tandem mass spectrometry [LC-MS/MS]), as well as patient information obtained from the clinic (i.e., C-reactive protein [CRP] levels, sex, age, smoking history, Disease Activity Score [DAS] and Health Assessment Questionnaire [HAQ] scores). In Fig. 3a, a PCA model (*R* ^2^ = 0.33, *Q* ^2^ = 0.17) of the data is presented. It is evident that the majority of the patients with no response to treatment (*dark circles*) cluster in one part of the plot. This clustering indicates that there are features within the model that have a predictive value for future response to treatment. However, there are also patients who did respond to treatment found within this cluster. To distinguish which factors most prominently separated the response to MTX from no response to MTX, an OPLS-DA model of the data was created (*R* ^2^ = 0.28, *Q* ^2^ = 0.17, CV-ANOVA *p* value = 0.01). In Fig. 2b, the features of this model that correlate with 95% confidence with the respective patient group are shown. Particularly, the Fc galactosylation factors are very prominent, with the most robust marker (signified by the top ranking and small error bar) being the ratio between the main agalactosylated and mono- and di-galactosylated Fc glycans of IgG1, (i.e., FA2/[FA2G1 + FA2G2]). In addition to the Fc galactosylation status, several of the complement proteins (complement factors I and H as well as complements 5 and 9) were ranked higher than the inflammatory marker CRP.

### Baseline FA2/(FA2G1 + FA2G2) of IgG1 can predict response to MTX treatment in early RA

**Fig. 4:**
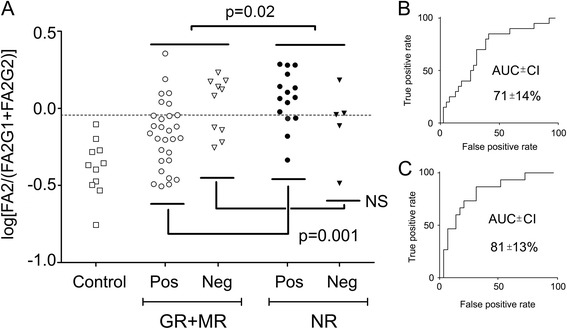
The log[FA2/(FA2G1 + FA2G2)] value of immunoglobulin G1 (IgG1) as a predictive factor for response. a Good (GR) and moderate responders (MR) compared with patients with no response (NR) to methotrexate treatment. Patients are split into seropositive (Pos) and seronegative (Neg) groups. Cutoff −0.045. b ROC curve for distinguishing the responding and nonresponding patient cohorts. c ROC curve for distinguishing the responding and nonresponding patient cohorts when only seropositive patients were included. Following false discovery rate correction, p = 0.001 remains significant. FA2 Main agalactosylated, FA2G1 Main monogalactosylated Fc glycan, FA2G2 Main digalactosylated Fc, NS Not significant

Because uni- and multivariate data analyses indicated that the baseline Fc glycan ratio of FA2/(FA2G1 + FA2G2) of IgG1 was the top candidate for distinguishing between MTX responders and nonresponders, we further investigated this marker’s performance in predicting therapeutic response (Table 3). We created a dichotomous biomarker choosing a cutoff (−0.045) of log[FA2/(FA2G1 + FA2G2)] (Fig. 4a) for optimal sensitivity and specificity determined by ROC curve of the model based on all patients (Fig. 4b). FA2/(FA2G1 + FA2G2) of IgG1 was significantly associated with lack of response among all patients (OR 5.3, 95% CI 1.6–17.0, *p* = 0.006). At the cutoff of −0.045, this parameter distinguished responders from nonresponders with a sensitivity of 70% (95% CI 46–88%) and specificity of 69% (95% CI 52–83%) (Table 3).

**Table 3:** Predictive value of immunoglobulin G1 log_10_[FA2/(FA2G2 + FA2G2)] as a response biomarker^a^

Test		Result	NR	GR + MR	Sensitivity (nonresponse) (95% CI)	Specificity (response) (95% CI)	OR (95% CI)
All patients	FA2/(FA2G2 + FA2G2)-IgG1	Positive	14	12	70% (46–88)	69% (52–83)	5.3 (1.6–17.0)
		Negative	6	27			
ACPA+ and/or RF+	FA2/(FA2G2 + FA2G2)-IgG1	Positive	11	6	73% (45–92)	79% (60–92)	10.5 (2.5–45)
		Negative	4	23			

*Abbreviations*: *ACPA* Anticyclic citrullinated peptide antibody, *FA2* Main agalactosylated, *FA2G1* Main monogalactosylated Fc glycan, *FA2G2* Main digalactosylated Fc, *GR* Good response, *IgG1* Immunoglobulin G1, *MR* Moderate response, *NR* No response, *RF* Rheumatoid factor

^a^Cutoff −0.045

Noteworthy among seropositive patients (i.e., patients who at baseline were testing positive for ACPA and/or RF, *n* = 44) is that the marker was even more selective (OR 10.5, 95% CI 2.5–45, *p* = 0.001). Responding patients could be predicted with a sensitivity of 73% (95% CI 45–92%) and a specificity of 79% (95% CI 60–92%) according to the −0.045 cutoff (Table 3). ROC curve analysis of seropositive patients generated an AUC of 81 ± 13% compared with the ROC curve model based on all patients (71 ± 14% for all patients) (Fig. 4).

Using logistic regression models, we identified significant association of lack of response with age (*p* = 0.03), whereas other tested variables (DAS score based on erythrocyte sedimentation rate [ESR], CRP levels, HAQ scores, sex, smoking status, use of prednisolone) were not significant predictors. When we included age as a possible predictor in the model, we observed that the FA2/(FA2G1 + FA2G2)-IgG1 biomarker remained a significant predictive factor associated with lack of response (all patients, OR 4.2, 95% CI 1.2–14.1, *p* = 0.02) and seropositive patients (OR 8.8, 95% CI 1.9–41.2, *p* = 0.006), respectively).

## Discussion

The glycosylation pattern of the IgG-Fc glycans has been associated with inflammation and immune activation in RA [8–10]. We confirm a changed galactosylation pattern of Fc glycans in RA that is partially reversed following MTX treatment [16, 18, 25]. We further show that specific Fc glycans (i.e., the ratio between the most abundant agalactosylated glycan versus the most abundant mono- and di-galactosylated Fc glycans of IgG1 [FA2/{FA2G1 + FA2G2}]) are associated with and have predictive value for MTX response. This result suggests a potential use of this marker in the clinic.

We used a previously validated sensitive proteomic approach [23] that enables quantification of a larger number of Fc glycans and distinguishes different IgG isotypes without any need for IgG enrichment. Given that protein G and protein A columns are based on Fc binding interactions, the regular “high-throughput” IgG enrichment approaches may affect the distribution of which Fc glycans are enriched. In addition, our method has the benefit of acquiring information on the regular proteome during the same analysis. This information can be used as an additional internal validation (as demonstrated by the complement pathway and glycan correlations presented herein). Thus, this approach provides a descriptive and direct measure of the polyclonal IgG-Fc glycan structures combined with the information on the regular proteome status [23]. A disadvantage of not including an IgG enrichment step is that the low abundant Fc glycopeptides, such as the IgG3/IgG4 Fc glycans, are below the limit of quantification. Additionally, owing to the complexity of the sample, the LC-MS/MS analysis time will be longer than the regular high-throughput approaches (1–2 h versus 5–30 minutes).

The altered Fc glycosylation pattern in RA is due mainly to the generally higher abundance of the agalactosylated glycans than that of their galactosylated counterparts. In the present study, we found that this perturbation in the Fc glycan profile occurs not only in IgG1 but also in IgG2 and that MTX treatment partially restores both profiles. Restoration of Fc galactosylation following treatment has been indicated in two previous RA studies [16, 18]. This change is likely a reflection and a marker of an overall decline in ongoing proinflammatory pathological processes following treatment. For example, a decline in IgG-Fc galactosylation has previously been associated with a proinflammatory status and correlates with CRP or ESR levels [9, 10, 12, 16]. Similarly to other reports in the literature, we also found a correlation between IgG1 [FA2/(FA2G1 + FA2G2)] and CRP (*R* ^2^ = 0.3 both prior to and following treatment). However, in contrast to FA2/(FA2G1 + FA2G2)-IgG1, baseline DAS measured by DAS28-ESR and baseline CRP levels were not significantly associated with response, suggesting that the IgG galactosylation patterns in RA likely reflect complex disease processes beyond inflammation. Furthermore, the DAS correlated only with IgG1 [FA2/(FA2G1 + FA2G2)] following treatment (*R* ^2^ = 0.2); at baseline, the trend was insignificant (*R* ^2^ = 0.02).

Of the 59 patients with RA, 15 (6 with good, 6 with moderate, and 3 with no MTX response) were treated with prednisolone. Corticosteroids such as prednisolone have been shown to affect protein glycosylation, particularly sialylation [29–31]. In the present study, we could not see an effect of prednisolone on sialylation levels or galactosylation status. When we excluded the prednisolone-treated patients, log(FA2/(FA2G1 + FA2G2) remained significant (*p* < 0.03).

Because Fc galactosylation status has been linked to both regulation of the lectin complement pathway (via agalactosylated Fc glycans) [3] and the classical complement pathway (via galactosylated Fc glycans) [32, 33], we tested how these proteins (also analyzed in the same sera LC-MS/MS measurements) would correlate with the galactosylation status. It is noteworthy that, in the multivariate model, both proteins that were highest-ranked in distinguishing responding and nonresponding patients and that could be linked to a specific pathway were regulatory proteins of the alternative complement pathway (CFI and CFH). Additionally, the membrane attack complex-forming proteins (C5 and C9) strongly correlated with no response to MTX, suggesting a high level of proinflammatory activity in these patients. Note that both the classical pathway proteins C1qb and C1qc had significantly lower abundance in patients with RA than in control subjects (Additional file 1: Figure S4). This is in line with previous studies that have demonstrated a positive correlation between Fc galactosylation and classical complement pathway activation via C1q binding [32, 33]. It is noteworthy that the protein which showed the best intraindividual correlation with galactosylation status (including control subjects and patients with RA) was the inhibitor of the classical and lectin complement pathways C4bBPα, whose abundance was positively correlated with agalactosylation (Additional file 1: Figure S5).

By investigating the discriminatory value of several different Fc glycans, we found that FA2/(FA2G1 + FA2G2) of IgG1 was the best predictive factor for therapeutic response to MTX. Interestingly, among seropositive (ACPA- and/or RF-positive) patients, this trend was enhanced, suggesting a potential pathogenic relevance of the glycosylation status of RA-associated antibodies similar to what has previously been proposed [2, 11, 12, 34]. However, this observation needs to be confirmed and validated in a larger cohort.

Researchers in one previous study on patients with long-standing RA treated with MTX or anti-tumor necrosis factor drugs tested the use of Fc galactosylation status as a biomarker for predicting MTX response [16]. In that study, the results were negative. Besides the clinical difference between the cohorts (early RA versus long-standing RA, naive versus previous exposure to disease-modifying antirheumatic drugs), another possible explanation for this discrepancy might rely on the differences in analytical techniques. This previous report was limited to the mono-galactosylated glycan abundance (FAG1) from unspecific IgGs [16], whereas in the present study, we found more prominent differences for the di-galactosylated counterpart (FA2G2) than for FA2G1.

## Conclusions

In this pilot, we show that the IgG-Fc galactosylation status is changed in untreated patients with early RA, which likely has perturbing effects on activation and regulation of the complement pathways. Furthermore, FA2/(FA2G1 + FA2G2) of IgG1 at baseline is significantly associated with nonresponse and thus is a potential biomarker for MTX clinical response prediction. The overall results, and particularly the specific differences in predicting the MTX response comparing seropositive and seronegative patients, are interesting but need to be validated in larger cohorts.

## Additional file

Additional file 1: Table S1. Demographic characteristics of the nationwide EIRA cohort. **Table S2.** Characterized complement pathway proteins and IgG-isotype proteins. **Table S3.** Individual IgG-Fc glycan distribution values in IgG1 and in IgG2 in control subjects, as well as in patients with RA prior to and following MTX treatment. **Table S4.** Significant differences for the 19 characterized glycan species when comparing healthy versus all, good, moderate, and nonresponding patients prior to and following MTX treatment. **Table S5.** IgG1 and IgG2 Fc glycan distributions grouped according to structural features, comparing healthy control subjects and patients with RA prior to and following MTX treatment. **Table S6.** Intra- and interindividual differences in the patients with RA, comparing individual glycan species prior to and following MTX treatment. **Table S7.** Complete list, ranking, and correlation (with response versus no response to MTX) of the features used in the OPLS-DA model shown in Fig. 3b. **Figure S1.** Extracted ion chromatograms of IgG1 and IgG2 Fc glycans quantified in a control subject and a patient with RA. **Figure S2.** Intraindividual changes in galactosylation status on IgG1 and on IgG2 for good and moderate responders and for nonresponders. **Figure S3.** Intraindividual correlation between the aGal/Gal status of glycans with different types of structural features and of IgG1 and IgG2 substituted glycans. **Figure S4.** Significant differences between control subjects and patients with early RA at baseline in the classical pathway initiating complements C1 and C9. **Figure S5.** Intraindividual correlation between the classical and lectin pathway inhibitor C4bBPα versus FA2/(FA2G1 + FA2G2). (DOCX 1180 kb)

## Abbreviations

ACPA: Anticitrullinated protein antibodies
aF: Lacking fucose
aG: Lacking galactose
aGal/Gal: Ratio between agalactosylated and galactosylated glycans
B: Bisected glycans
C: Complement protein
CF: Complement factor
CRP: C-reactive protein
CV-ANOVA: Analysis of variance of cross-validated residuals
DAS-ESR: Disease Activity Score based on erythrocyte sedimentation rate
EIRA: Epidemiological Investigation of Rheumatoid Arthritis
EULAR: European League Against Rheumatism
FA2: Main agalactosylated Fc glycan
FA2G1: Main monogalactosylated Fc glycan
FA2G2: Main digalactosylated Fc glycan
Fc: Fragment crystallizable
FDR: False discovery rate
G: Galactose
GR: Good response
HAQ: Health Assessment Questionnaire
IgG: Immunoglobulin G
MR: Moderate response
MS: Mass spectrometer
MS/MS: Tandem mass spectrometry
LC-MS/MS: Liquid chromatography-tandem mass spectrometry
MTX: Methotrexate
NR: No response
OPLS-DA: Orthogonal projections to latent structures discriminant analysis
PCA: Principal component analysis
RA: Rheumatoid arthritis
RF: Rheumatoid factor
S: Sialic acid
