# Supplementary material for: IgG Fc galactosylation predicts response to methotrexate in early rheumatoid arthritis
Source: Arthritis Res Ther. 2017 Aug 9;19:182. doi: 10.1186/s13075-017-1389-7 (PMC5549282; doi:10.1186/s13075-017-1389-7)

**Additional file 1**

**Tables**

**Table S1.** Demographic characteristics of the nationwide EIRA cohort.

**Table S2.** Characterized complement pathway proteins and IgG-isotype proteins.

**Table S3.** Individual IgG-Fc glycan distribution values in IgG_1_ and in IgG_2_ in Controls, as well as in RA patients prior to and following MTX treatment.

**Table S4.** Significant differences for the 19 characterized glycan species when comparing Healthy *versus* All, Good, Moderate and Non-responding patients prior to and following MTX treatment.

**Table S5.** IgG_1_ and IgG_2_ Fc-glycan distributions grouped according to structural features, when comparing healthy controls and RA patients prior to and following MTX treatment.

**Table S6.** Intra- and inter-individual differences in the RA patients when comparing individual glycan species prior to and following MTX treatment.

**Table S7.** Complete list, ranking and correlation (with response *versus* no response to MTX), of the features used in the OPLS-DA model shown in Figure 3B.

**Figures**

**Figure S1.** Extracted ion chromatograms of IgG_1_ and IgG_2_ Fc-glycans quantified in a control and a RA patient.

**Figure S2.** Intra-individual changes in galactosylation status on IgG_1_ and on IgG_2_ for good, moderate and non-responders.

**Figure S3.** Intra-individual correlation between the aGal/Gal status of glycans with different types of structural features and of IgG_1_ and IgG_2_ substituted glycans.

**Figure S4.** Significant differences between controls and early RA patients at baseline in the classical pathway initiating complement C1 and C9.

**Figure S5.** Intra-individual correlation between the classical and lectin pathway inhibitor C4bBPα *versus* FA2/(FA2G1+FA2G2).

**Table S1. Demographic characteristics of the nationwide EIRA cohort.**

|  | **EIRA-MTX-glycan study** | **EIRA-MTX-Solna** | **EIRA I-mono MTX Sweden** |
| --- | --- | --- | --- |
| **Number of individuals** | 59 | 183 | 842 |
| **Age at inclusion, year** | 53 (45-62) | 52 (42-59) | 55 (45-62) |
| **Female sex** | 42 (71%) | 132 (72%) | 69% |
| **Ever smoking** | 29 (59%) | 107 (66%) | 57% |
| **HLA-SE** | 44 (75%) | 143 (79%) | 75% |
| **Anti-CCP2 positive** | 39 (66%) | 125 (68%) | 65% |
| **RF positive** | 41 (69%) | 124 (68%) | 67% |
| **DAS28-ESR** | 5.7 (5.0-6.2) | 5.7 (5.1-6.2) | 5.6 (4.8-6.3) |
| **3 month EULAR good response** | 19 (32%) | 52 (28%) | 32% |
| **Prednisolone** | 15 (25%) | 26 (15%) | 31% |

**Table S2.** **Characterized complement pathway proteins and IgG-isotype proteins.** Table contains the number of unique peptides used to quantify respective protein and their corresponding peptide median scores.

| **Protein** | **Number of peptides** | **Median score** |
| --- | --- | --- |
| C1qB^a^ | 5 | 44 |
| C1qC^b^ | 4 | 32 |
| C1r^c^ | 4 | 39 |
| C1s^d^ | 10 | 68 |
| C2^e^ | 2 | 33 |
| C3^f^ | 162 | 47 |
| C5^g^ | 41 | 37 |
| C6^h^ | 17 | 39 |
| C7^i^ | 15 | 37 |
| C8α^j^ | 10 | 50 |
| C8β^k^ | 14 | 29 |
| C8γ^l^ | 8 | 58 |
| C9^m^ | 22 | 54 |
| CFB^n^ | 33 | 48 |
| CFH^o^ | 30 | 48 |
| CFHrp1^p^ | 3 | 29 |
| CFHrp2^q^ | 3 | 35 |
| CFI_LC^r^ | 14 | 42 |
| C4b-BPα^s^ | 19 | 48 |
| C4b-BPβ^t^ | 2 | 28 |
| IgG_1_ | 8 | 63 |
| IgG_2_ | 7 | 48 |
| IgG_3_ | 5 | 58 |
| IgG_4_ | 6 | 63 |

^a^Complement C1q subcomponent subunit B, ^b^Complement C1q subcomponent subunit C, ^c^Complement C1r subcomponent, ^d^Complement C1s subcomponent, ^e^Complement C2, ^f^Complement C3, ^g^Complement C5, ^h^Complement C6, ^i^Complement C7, ^j^Complement C8 alpha chain, ^k^Complement C8 beta chain, ^l^Complement C8 gamma chain, ^m^Complement C9, ^n^Complement factor B, ^o^Complement factor H, ^p^Complement factor H-related protein 1, ^q^Complement factor H-related protein 2, ^r^Complement factor I light chain, ^s^Complement C4b-binding protein alpha chain, ^t^Complement C4b-binding protein beta chain

**Table S3. Individual IgG-Fc glycan distribution values in IgG_1_ and in IgG_2_ in Controls, as well as in RA patients prior to and following MTX treatment.** Data is from all patients combined as well as according to good, moderate and no response to MTX treatment. Values are given in average distribution (%) ± standard deviation.

|  | Isotype | Glycan^a^ | Control | RA baseline | | RA MTX treated | Good responders | | | Moderate responders | | | | | Non responders | | | |
| --- | --- | --- | --- | --- | --- | --- | --- | --- | --- | --- | --- | --- | --- | --- | --- | --- | --- | --- |
|  |  |  |  |  |  | | Prior | Following | | | Prior | | Following | | | Prior | | Following |
| Individual glycans | IgG_1_ | A2 | 0.8±0.9 | 0.4±0.8 | | 0.5±0.9 | 0.8±1.2 | 0.8±1.5 | 0.3±0.3 | | | 0.4±0.4 | | 0.2±0.2 | | | 0.4±0.5 | |
|  |  | A2B | 0.6±0.4 | 1.8±1.2 | | 2.0±1.6 | 1.5±1.0 | 1.3±0.9 | 2.0±1.4 | | | 2.0±1.6 | | 2.0±1.3 | | | 2.6±2.1 | |
|  |  | A2G1 | 2.0±1.4 | 0.8±1.0 | | 0.8±1.1 | 1.1±1.1 | 1.1±1.1 | 0.6±1.0 | | | 0.8±1.0 | | 0.5±0.7 | | | 0.5±0.6 | |
|  |  | A2G2 | 0.8±0.8 | 0.2±0.6 | | 0.3±0.6 | 0.3±0.4 | 0.4±0.5 | 0.3±0.9 | | | 0.3±0.8 | | 0.1±0.3 | | | 0.2±0.2 | |
|  |  | FA2 | 24±7 | 38±11 | | 36±12 | 33±10 | 30±10 | 39±11 | | | 35±11 | | 43±10 | | | 44±12 | |
|  |  | FA2B | 4±1 | 5±2 | | 5±2 | 5±2 | 5±3 | 5±2 | | | 5±2 | | 6±2 | | | 6±3 | |
|  |  | FA2G1 | 38±3 | 34±5 | | 33±5 | 36±5 | 36±4 | 34±5 | | | 34±5 | | 32±4 | | | 31±6 | |
|  |  | FA2BG1 | 8±2 | 5±2 | | 6±2 | 6±2 | 7±2 | 5±2 | | | 6±2 | | 5±2 | | | 5±2 | |
|  |  | FA2G1S1 | 0.1±0.1 | 0.2±0.2 | | 0.2±0.2 | 0.2±0.2 | 0.2±0.2 | 0.2±0.2 | | | 0.2±0.1 | | 0.2±0.2 | | | 0.2±0.2 | |
|  |  | FA2G2 | 18±5 | 11±5 | | 12±6 | 13±5 | 15±7 | 11±5 | | | 12±5 | | 9±5 | | | 8±5 | |
|  |  | FA2BG2 | 0.6±0.6 | 0.2±0.3 | | 0.3±0.4 | 0.3±0.2 | 0.4±0.4 | 0.3±0.4 | | | 0.3±0.4 | | 0.1±0.2 | | | 0.1±0.2 | |
|  |  | FA2G2S1 | 4±2 | 3±2 | | 3±3 | 4±3 | 4±3 | 3±2 | | | 3±2 | | 2±2 | | | 2±2 | |
|  | IgG_2_ | FA2 | 38±11 | 49±13 | | 48±13 | 42±12 | 41±13 | 50±12 | | | 47±11 | | 55±11 | | | 55±11 | |
|  |  | FA2B | 6±2 | 5±2 | | 6±3 | 5±3 | 5±3 | 5±2 | | | 5±2 | | 6±2 | | | 6±3 | |
|  |  | FA2G1 | 36±4 | 32±7 | | 32±6 | 35±6 | 35±5 | 32±7 | | | 33±6 | | 29±6 | | | 29±7 | |
|  |  | FA2BG1 | 4±2 | 2±1 | | 2±1 | 2±1 | 3±1 | 1±1 | | | 2±1 | | 1±1 | | | 1±1 | |
|  |  | FA2G1S1 | 1±1 | 2±1 | | 2±1 | 2±1 | 2±1 | 2±1 | | | 1±1 | | 1±1 | | | 2±1 | |
|  |  | FA2G2 | 12±6 | 7±5 | | 8±5 | 9±5 | 11±6 | 7±4 | | | 8±4 | | 5±4 | | | 5±2 | |
|  |  | FA2G2S1 | 4±3 | 3±3 | | 3±3 | 4±4 | 4±4 | 3±2 | | | 3±3 | | 2±2 | | | 2±2 | |

^a^Glycan abbreviations are provided in Figure 2A.

**Table S4.** **Significant differences for the 19 characterized glycan species when comparing Healthy *versus* All, Good, Moderate and Non-responding patients prior to and following MTX treatment.** Good responding (GR), moderately responding (MR) and no responding (NR) patients. P-values below 0.02 remain significant following FDR correction.

|  |  |  | **Controls vs RA Prior MTX treatment** | | | | **Controls vs RA Following MTX treatment** | | | |
| --- | --- | --- | --- | --- | --- | --- | --- | --- | --- | --- |
|  | Isotype | Glycan^a^ | Total | GR | MR | NR | Total | GR | MR | NR |
| Individual glycoforms | IgG_1_ | A2 | 0.2 | 1.0 | 0.1 | 0.1 | 0.4 | 0.9 | 0.2 | 0.2 |
|  |  | A2B | **0.0000001** | **0.004** | **0.003** | **0.0001** | **0.000001** | **0.01** | **0.001** | **0.001** |
|  |  | A2G | **0.001** | 0.1 | **0.02** | **0.01** | **0.03** | 0.1 | **0.01** | **0.01** |
|  |  | A2G2 | **0.01** | 0.1 | 0.1 | **0.02** | **0.02** | 0.1 | 0.1 | **0.02** |
|  |  | FA2 | **0.0001** | **0.01** | **0.0001** | **0.000005** | **0.002** | 0.1 | 0.004 | **0.00002** |
|  |  | FA2B | 0.1 | 0.2 | 0.2 | **0.01** | **0.01** | 0.2 | 0.1 | **0.01** |
|  |  | FA2G1 | **0.003** | 0.1 | **0.001** | **0.0003** | **0.0002** | 0.1 | 0.01 | **0.00003** |
|  |  | FA2BG1 | **0.001** | **0.02** | **0.01** | **0.001** | **0.03** | 0.2 | 0.1 | **0.01** |
|  |  | FA2G1S1 | **0.0001** | **0.02** | **0.02** | **0.003** | **0.001** | 0.1 | 0.02 | **0.01** |
|  |  | FA2G2 | **0.0002** | **0.03** | **0.002** | **0.00003** | **0.005** | 0.2 | 0.01 | **0.0001** |
|  |  | FA2BG2 | 0.1 | 0.1 | 0.1 | **0.02** | 0.1 | 0.2 | 0.1 | **0.02** |
|  |  | FA2G2S1 | 0.2 | 0.7 | 0.2 | **0.02** | 0.4 | 1.0 | 0.5 | **0.04** |
|  | IgG_2_ | FA2 | **0.01** | 0.3 | **0.01** | **0.0004** | **0.02** | 0.5 | 0.03 | **0.0001** |
|  |  | FA2B | 0.6 | 0.6 | 0.4 | 1.0 | 0.9 | 0.8 | 0.8 | 0.9 |
|  |  | FA2G1 | **0.004** | 0.4 | **0.02** | **0.002** | 0.1 | 0.4 | 0.1 | **0.004** |
|  |  | FA2BG1 | **0.01** | **0.02** | **0.001** | **0.004** | **0.02** | 0.1 | 0.01 | **0.003** |
|  |  | FA2G1S1 | 0.3 | 0.1 | 0.3 | 0.8 | 0.7 | 0.4 | 0.8 | 0.7 |
|  |  | FA2G2 | **0.01** | 0.3 | **0.02** | **0.001** | **0.03** | 0.7 | 0.05 | **0.002** |
|  |  | FA2G2S1 | 0.3 | 0.8 | 0.3 | 0.1 | 0.4 | 1.0 | 0.6 | 0.1 |

^a^Glycan abbreviations are provided in Figure 1A

**Table S5. IgG_1_ and IgG_2_ Fc-glycan distributions grouped according to structural features, when comparing healthy controls and RA patients prior to and following MTX treatment.** Values are given in average distribution (%) ± standard deviation. P-values below 0.02 remain significant following FDR correction.

| Type | Isotype | Glycan^a^ | Healthy controls | Early RA patients | | Inter-individual differences | |
| --- | --- | --- | --- | --- | --- | --- | --- |
|  |  |  |  | Prior | Following | HC/ patient prior treatment | HC/ patient following treatment |
|  | IgG_1_ | Σ[galactosylated-glycans]^b^ | 71±8 | 54±13 | 56±15 | **0.0001** | **0.002** |
|  |  | Σ[agalactosylated-glycans]^c^ | 29±8 | 46±13 | 44±15 | **0.0001** | **0.002** |
|  |  | Σ[afucosylated-glycans]^d^ | 4±3 | 3±2 | 4±2 | 0.2 | 0.5 |
|  |  | Σ[bisected-glycans]^e^ | 13±3 | 13±3 | 14±4 | 0.9 | 0.5 |
|  |  | Σ[sialylated-glycans]^f^ | 4±2 | 3±2 | 3±3 | 0.2 | 0.5 |
|  | IgG_2_ | Σ[galactosylated-glycans]^g^ | 57±12 | 46±14 | 47±14 | **0.02** | **0.03** |
|  |  | Σ[agalactosylated-glycans]^h^ | 43±12 | 54±14 | 53±14 | **0.02** | **0.03** |
|  |  | Σ[bisected-glycans]^i^ | 9±3 | 7±3 | 7±3 | **0.01** | 0.1 |
|  |  | Σ[sialylated-glycans]^j^ | 5±4 | 5±4 | 5±4 | 0.7 | 0.6 |
| aGal/Gal ratio | All IgG_1_ | Log [Σ[agalactosylated-glycans] /Σ[galactosylated-glycans]] | -0.40±0.19 | -0.08±0.24 | -0.11±0.06 | **0.0001** | **0.002** |
|  | All IgG_2_ | Log [Σ[agalactosylated-glycans] /Σ[galactosylated-glycans]] | -0.12±0.22 | 0.08±0.25 | 0.06±0.26 | **0.02** | **0.03** |

Abbreviations good responders (GR), moderate responders (MR) and no responders (NR). ^a^Glycan abbreviations are provided in Figure 2A, ^b^Σ[IgG_1_:A2G1, A2G2, FA2G1, FA2BG1, FA2G1S1, FA2G2, FA2BG2, FA2G2S1], ^c^Σ[IgG_1_: FA2, FA2B, A2 and A2B]; ^d^Σ[IgG_1_:A2, A2B, A2G1, A2G2], ^e^Σ[IgG_1_:A2B, FA2B, FA2BG1, FA2BG2], ^f^Σ[IgG_1_:FA2G1S1, FA2G2S1], ^g^Σ[IgG_2_: FA2G1, FA2BG1, FA2G2, FA2G1S1, FA2G2, FA2G2S1], ^h^Σ[IgG_2_: FA2, FA2B]; ^i^Σ[IgG_2_: FA2B, FA2BG1], ^j^Σ[IgG_2_: FA2G1S1, FA2G2S1].

**Table S6.** **Table S6. Intra- and inter-individual differences in the RA patients when comparing individual glycan species prior to and following MTX treatment.** Good response (GR), moderate response (MR) and no response (NR) to treatment. P-values below 0.02 remain significant following FDR correction.

|  |  |  | Intra-individual change | | |  | | Inter-individual differences | |  |  |  |  | |  | | | |
| --- | --- | --- | --- | --- | --- | --- | --- | --- | --- | --- | --- | --- | --- | --- | --- | --- | --- | --- |
|  |  |  | Prior/Following | |  | |  | Prior |  |  | |  | | Following | | |  |  |
|  | Isotype | Glycan^a^ | Total | GR | MR | | PR | GR/NR | (GR+MR)/NR | MR/GR | | MR/NR | | GR/NR | | (GR+MR)/NR | MR/GR | MR/NR |
| Individual glycans | IgG_1_ | A2 | **0.03** | 0.5 | 0.2 | | 0.1 | 0.1 | 0.1 | 0.1 | | 0.5 | | 0.2 | | 0.3 | 0.2 | 1.0 |
|  |  | A2B | 0.5 | 0.4 | 0.9 | | 0.3 | 0.2 | 0.5 | 0.1 | | 0.9 | | **0.02** | | 0.1 | 0.1 | 0.4 |
|  |  | A2G1 | 0.2 | 0.9 | **0.05** | | 0.7 | **0.03** | 0.1 | 0.1 | | 0.6 | | **0.04** | | 0.1 | 0.3 | 0.3 |
|  |  | A2G2 | **0.04** | **0.04** | 0.8 | | 0.4 | 0.2 | 0.2 | 0.8 | | 0.4 | | **0.03** | | 0.1 | 0.7 | 0.4 |
|  |  | FA2 | **0.003** | **0.001** | **0.003** | | 0.8 | **0.003** | **0.01** | 0.1 | | 0.2 | | **0.0003** | | **0.0004** | 0.1 | **0.02** |
|  |  | FA2B | 0.1 | 0.8 | **0.01** | | 0.7 | 0.2 | 0.1 | 0.8 | | 0.1 | | 0.2 | | 0.2 | 0.6 | 0.3 |
|  |  | FA2G1 | 0.5 | 0.8 | 0.6 | | 0.1 | **0.03** | 0.1 | 0.2 | | 0.3 | | **0.003** | | **0.003** | 0.2 | 0.1 |
|  |  | FA2BG1 | **0.002** | **0.02** | **0.0002** | | 0.8 | 0.1 | 0.2 | 0.4 | | 0.4 | | **0.02** | | **0.01** | 0.7 | 0.1 |
|  |  | FA2G1S1 | 0.2 | 0.3 | 0.4 | | 0.6 | 0.5 | 0.5 | 0.9 | | 0.6 | | 0.3 | | 0.2 | 0.8 | 0.4 |
|  |  | FA2G2 | **0.02** | **0.02** | 0.1 | | 0.8 | **0.01** | **0.02** | 0.3 | | 0.1 | | **0.003** | | **0.004** | 0.2 | **0.04** |
|  |  | FA2BG2 | 0.1 | 0.1 | 0.9 | | 1.0 | **0.03** | **0.01** | 0.6 | | 0.1 | | **0.01** | | **0.002** | 0.4 | 0.1 |
|  |  | FA2G2S1 | 0.1 | 0.5 | 0.3 | | 0.5 | 0.1 | 0.1 | 0.4 | | 0.2 | | 0.1 | | 0.1 | 0.5 | 0.1 |
|  | IgG_2_ | FA2 | 0.1 | 0.1 | **0.02** | | 0.7 | **0.002** | **0.01** | 0.1 | | 0.2 | | **0.0004** | | **0.001** | 0.1 | **0.03** |
|  |  | FA2B | 0.1 | 0.2 | 0.1 | | 0.8 | 0.5 | 0.3 | 0.9 | | 0.3 | | 0.7 | | 0.7 | 1.0 | 0.7 |
|  |  | FA2G1 | 0.3 | 1.0 | 0.1 | | 1.0 | **0.01** | **0.04** | 0.1 | | 0.3 | | **0.01** | | **0.01** | 0.3 | 0.1 |
|  |  | FA2BG1 | **0.004** | **0.01** | **0.003** | | 0.7 | 0.1 | 0.4 | 0.1 | | 1.0 | | **0.001** | | **0.01** | 0.1 | 0.1 |
|  |  | FA2G1S1 | **0.04** | 0.3 | **0.02** | | 0.8 | 0.1 | 0.1 | 0.5 | | 0.3 | | 0.4 | | 1.0 | 0.2 | 0.4 |
|  |  | FA2G2 | 0.2 | **0.03** | 0.3 | | 0.3 | **0.01** | **0.01** | 0.2 | | 0.1 | | **0.001** | | **0.0001** | 0.1 | **0.01** |
|  |  | FA2G2S1 | 0.7 | 0.6 | 0.3 | | 1.0 | **0.02** | **0.02** | 0.2 | | 0.2 | | **0.05** | | **0.02** | 0.5 | 0.1 |

^a^Glycan acronyms are provided in Figure 1A

**Table S7. Complete list, ranking and correlation (with response *versus* no response to MTX), of the features used in the OPLS-DA model shown in Figure 3B.** The coordinates marked in gray were correlating with 95% confidence with either no-response (positive pq[1]) or with response (negative pq[1]).

| Factor | pq[1] | 95% confidence interval |
| --- | --- | --- |
| log(FA2/(FA2G1+FA2G2))-IgG_1_ | 0.18 | 0.05 |
| log(FA2b/(FA2bG1+FA2bG2))-IgG_1_ | 0.18 | 0.09 |
| log(FA2/(FA2G1+FA2G2))-IgG_2_ | 0.17 | 0.05 |
| CFI | 0.17 | 0.08 |
| log(A2/(A2G1+A2G2))-IgG_1_ | 0.16 | 0.08 |
| log(FA2b/(FA2bG1)) -IgG_2_ | 0.16 | 0.06 |
| CFH | 0.16 | 0.09 |
| C5 | 0.16 | 0.05 |
| C9 | 0.16 | 0.04 |
| C4b-binding protein α-chain | 0.15 | 0.10 |
| CRP | 0.15 | 0.07 |
| CFB | 0.15 | 0.06 |
| C6 | 0.14 | 0.06 |
| C8γ | 0.14 | 0.09 |
| C8β | 0.12 | 0.04 |
| C1r | 0.12 | 0.10 |
| Age | 0.12 | 0.06 |
| C8α | 0.12 | 0.05 |
| C3 | 0.12 | 0.06 |
| C1s | 0.11 | 0.14 |
| C1qB | 0.09 | 0.11 |
| CFH – related 1 | 0.07 | 0.05 |
| ∑Bisected - IgG_1_ | 0.07 | 0.09 |
| C2 | 0.07 | 0.06 |
| C1qC | 0.07 | 0.05 |
| C4b-binding protein β-chain | 0.06 | 0.10 |
| C7 | 0.06 | 0.05 |
| IgG_3_ | 0.05 | 0.15 |
| DAS | 0.05 | 0.05 |
| IgG_1_ | 0.04 | 0.10 |
| Smoker | 0.03 | 0.08 |
| HAQ | 0.03 | 0.06 |
| Male | 0.02 | 0.08 |
| CFH-related 2 | 0.02 | 0.12 |
| ∑Afucosylated - IgG_1_ | 0.02 | 0.14 |
| IgG_4_ | 0.02 | 0.08 |
| ∑Bisected - IgG_2_ | 0.01 | 0.17 |
| CCP positive | -0.02 | 0.05 |
| Female | -0.02 | 0.08 |
| RF positive | -0.04 | 0.10 |
| log(IgG_2_/IgG_4_) | -0.08 | 0.13 |
| IgG_2_ | -0.08 | 0.13 |
| ∑Sialylated -IgG_2_ | -0.10 | 0.10 |
| ∑Sialylated -IgG_1_ | -0.12 | 0.07 |
| ∑Gal -IgG_2_ | -0.16 | 0.08 |
| ∑Gal -IgG_1_ | -0.17 | 0.06 |

**Figure S1. Extracted ion chromatograms of IgG_1_ and IgG_2_ Fc-glycans quantified in a control and a RA patient.** In (A) a control and (B) a RA patient with no response to treatment. Note the prominently lower abundance of the sialylated (blue) and digalactosylated (green) species in the RA patient.

**
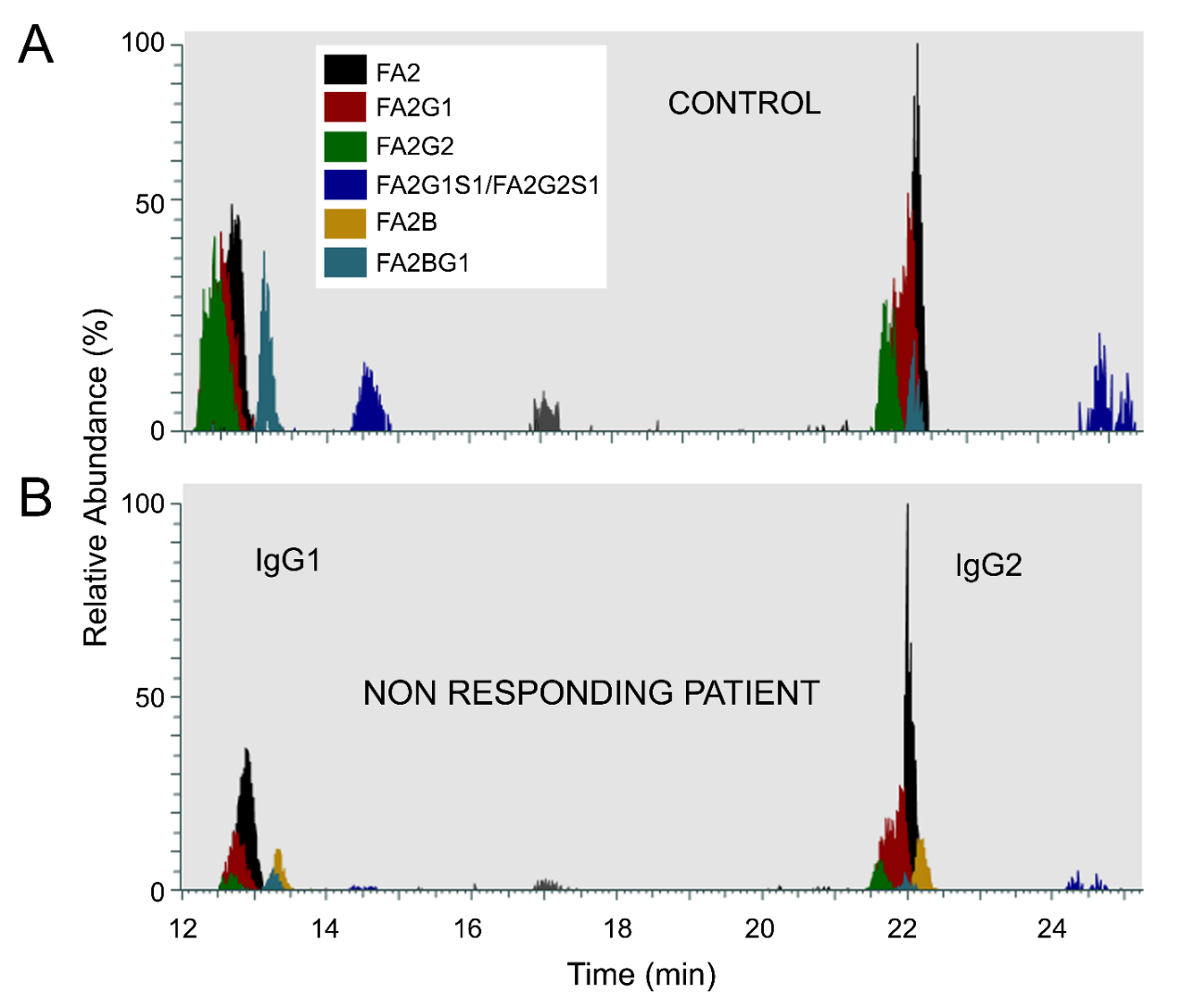
**

**Figure S2. Intra-individual changes in galactosylation status on IgG_1_ and on IgG_2_ for good, moderate and non-responders.** Following FDR correction p=0.004 remains significant.

**
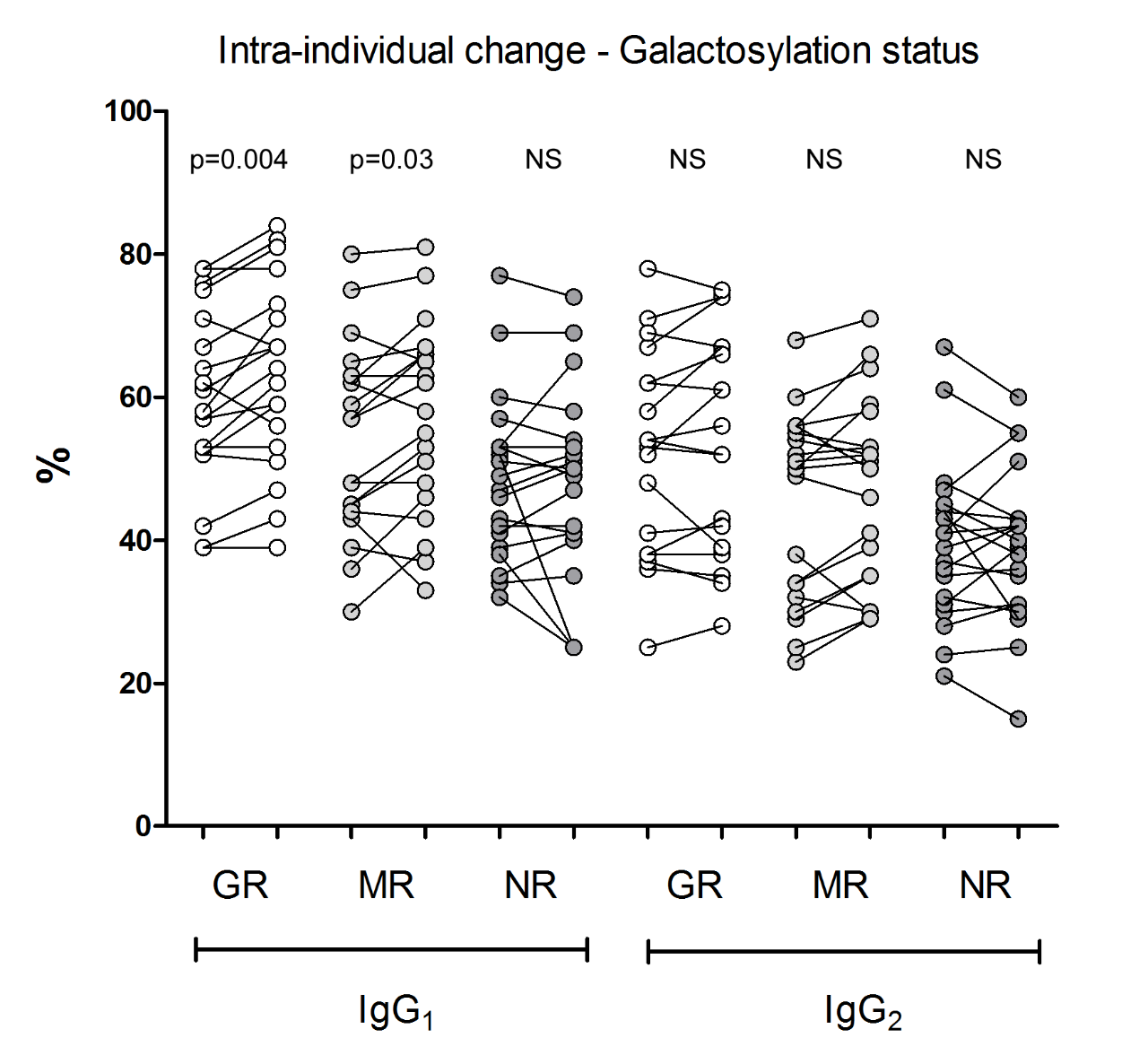
**

**Figure S3. Intra-individual correlation between the aGal/Gal status of glycans with different types of structural features and of IgG_1_ and IgG_2_ substituted glycans. (A)** The correlation between aGal/Gal of IgG_1_: log[FA2/(FA2G1+FA2G2)] vs log[FA2B/(FA2BG1+FA2BG2)] (red dots), IgG_1_: log[FA2/(FA2G1+FA2G2)] vs log[A2/(A2G1+A2G2)] (white dots) and IgG_2_: log[FA2/(FA2G1+FA2G2)] vs log[FA2B/FA2BG1] (pink dots). (**B**) The correlation between log[FA2/(FA2G1+FA2G2)] in IgG_1_ vs IgG_2_ (red dots) and of the bisected counterparts (pink dots).





**Figure S4. Significant differences between controls and early RA patients at baseline in the classical pathway initiating complement C1 and C9.** Patients are grouped according to their future response to MTX treatment (i.e. good response [GR], moderate response [MR] and no response [NR]). (**A**) C1qB, (**B**) C1qB, (**C**) C1r and (**D**) C9, respectively. P-values below 0.02 remain significant following FDR correction.





**Figure S5.** **Intra-individual correlation between the classical and lectin pathway inhibitor C4bBPα versus FA2/(FA2G1+FA2G2).** (**A**) FA2/(FA2G1+FA2G2) of IgG_1_ and (**B**) FA2/(FA2G1+FA2G2) of IgG_2_.
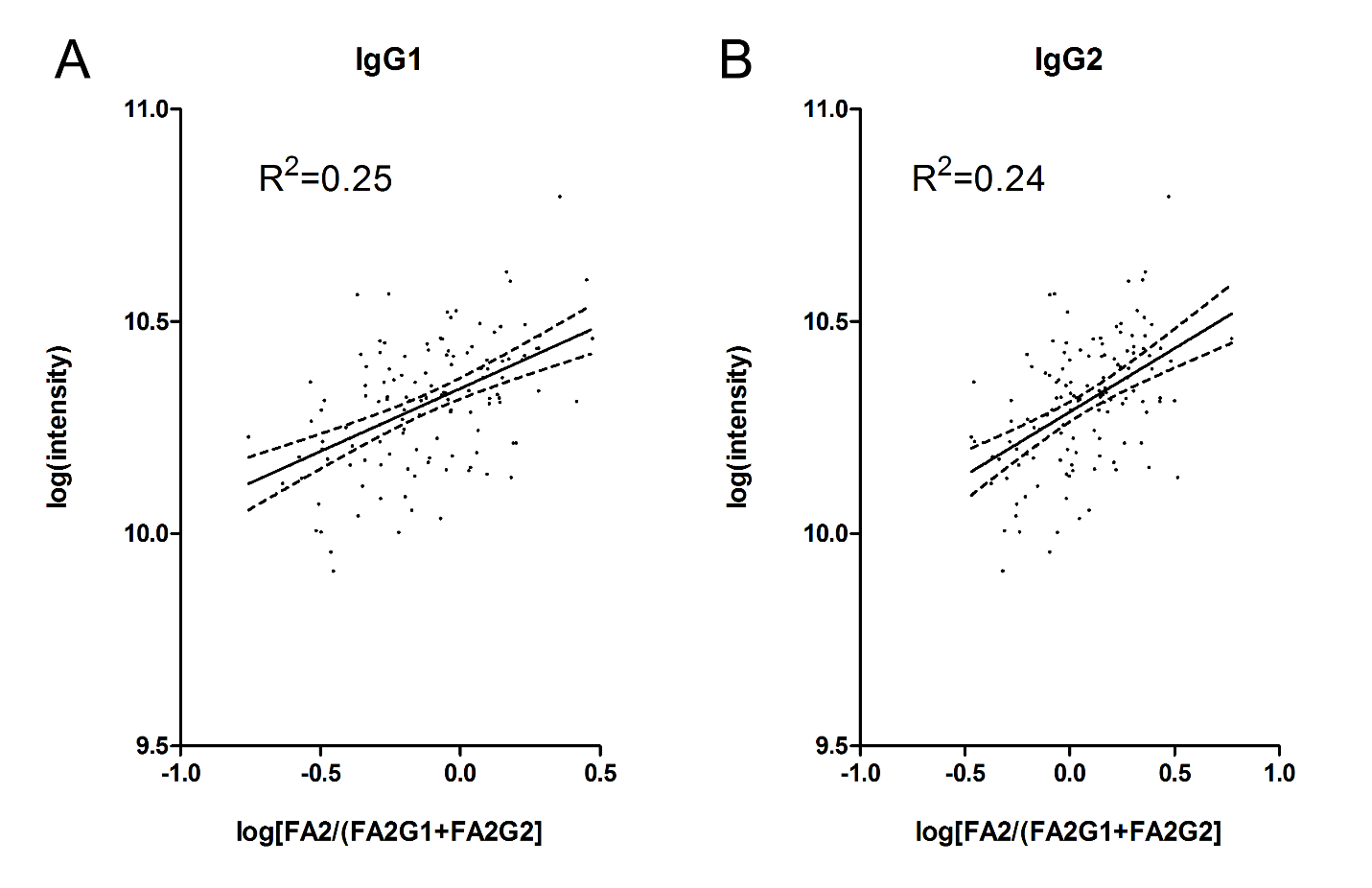

Supplement: Additional file 1: Table S1. — Demographic characteristics of the nationwide EIRA cohort. Table S2. Characterized complement pathway proteins and IgG-isotype proteins. Table S3. Individual IgG-Fc glycan distribution values in IgG1 and in IgG2 in control subjects, as well as in patients with RA prior to and following MTX treatment. Table S4. Significant differences for the 19 characterized glycan species when comparing healthy versus all, good, moderate, and nonresponding patients prior to and following MTX treatment. Table S5. IgG1 and IgG2 Fc glycan distributions grouped according to structural features, comparing healthy control subjects and patients with RA prior to and following MTX treatment. Table S6. Intra- and interindividual differences in the patients with RA, comparing individual glycan species prior to and following MTX treatment. Table S7. Complete list, ranking, and correlation (with response versus no response to MTX) of the features used in the OPLS-DA model shown in Fig. 3b. Figure S1. Extracted ion chromatograms of IgG1 and IgG2 Fc glycans quantified in a control subject and a patient with RA. Figure S2. Intraindividual changes in galactosylation status on IgG1 and on IgG2 for good and moderate responders and for nonresponders. Figure S3. Intraindividual correlation between the aGal/Gal status of glycans with different types of structural features and of IgG1 and IgG2 substituted glycans. Figure S4. Significant differences between control subjects and patients with early RA at baseline in the classical pathway initiating complements C1 and C9. Figure S5. Intraindividual correlation between the classical and lectin pathway inhibitor C4bBPα versus FA2/(FA2G1 + FA2G2). (DOCX 1180 kb) [file 13075_2017_1389_MOESM1_ESM.docx]
